# Clinical Features of Combined Central Retinal Artery and Vein Occlusion

**DOI:** 10.1155/2019/7202731

**Published:** 2019-10-09

**Authors:** Hao Wang, Yongye Chang, Fen Zhang, Rong Yang, Suxia Yan, Jieying Dong, Minglian Zhang, Shaomin Peng

**Affiliations:** ^1^Aier School of Ophthalmology, Central South University, Changsha 410015, China; ^2^Department of Ophthalmology, Hebei Eye Hospital, Xingtai 054001, China; ^3^Hebei Key Laboratory of Ophthalmology, Hebei Eye Hospital, Xingtai 054001, China; ^4^Department of Ophthalmology, Changde Xiangya Hospital, Changde 415000, China; ^5^Harbin Aier Eye Hospital, Harbin 150016, China

## Abstract

**Purpose:**

To describe the clinical features of combined central retinal artery and vein occlusion (CCRAVO).

**Methods:**

This retrospective study included 33 admitted patients (33 eyes) who had CCRAVO. Clinical data, such as age, gender, best-corrected visual acuity (BCVA), intraocular pressure (IOP), findings on fundus color photography and fundus fluorescein angiography (FFA), and information about follow-up, were collected and analyzed.

**Results:**

The age of the patients with CCRAVO ranged from 22 to 78 years, with a mean of 48.8 ± 14.1 years. At presentation, BCVA of the involved eyes ranged from no light perception (NLP) to 20/20. In addition, 45.5% (15/33) of the eyes had BCVA of finger counting (FC) or below, whereas 12.1% (4/33) had BCVA of 20/60 or above. The IOP was lower in the involved eyes than in the fellow eyes (15.0 ± 3.0 mmHg vs. 16.4 ± 2.3 mmHg, *p*=0.03). Ophthalmoscopic examination showed changes in both central retinal artery occlusion (CRAO) and central retinal vein occlusion (CRVO), including retinal hemorrhage, retinal ischemic whitening, optic disc hyperemia and/or edema, venous dilation and tortuosity, cotton wool spot (CWS), and Roth's spot. FFA showed prolonged arm-to-retina time (ART) and retinal arteriovenous passage time (RAP) (17.1 ± 4.9 s and 12.1 ± 8.8 s, respectively). Capillary nonperfusion (CNP) was seen in 21 eyes (63.6%), and in 14 (42.2%) of these, CNP was larger than 10 disc areas. At 2 to 3 weeks after presentation, BCVA improved in 23 eyes (71.9%) and further deteriorated in 5 eyes (15.6%). Retinal ischemic whitening improved in more than half of the eyes, whereas retinal hemorrhage increased in nearly half of the eyes. Follow-up ranged from 6 to 56 months. Seven patients were lost to follow-up. At final follow-up, six eyes had a visual acuity of 20/60 or greater, but 6 eyes had FC or worse. Four eyes developed neovascularization on follow-up.

**Conclusion:**

CCRAVO is a sight-threatening entity. Manifestations of CRAO and CRVO can be seen simultaneously in the early stage of disease, and CRVO may play a more important role in the development of CCRAVO.

## 1. Introduction

Combined central retinal artery and vein occlusion (CCRAVO) is an uncommon disorder that can cause severe visual damage. This entity combines the clinical features of central retinal artery occlusion (CRAO) and central retinal vein occlusion (CRVO), such as ischemic retinal whitening, a cherry-red spot in the macula, retinal hemorrhage, and dilated and/or tortuous retinal veins. Conditions such as systemic lupus erythematosus [1, 2], Behçet's syndrome [3], orbital inflammatory pseudotumor [4], posterior scleritis [5], retrobulbar injection [6], ocular trauma [7], hyperhomocysteinemia [8], and leukemia [9] have been reported to be associated with this disorder. Since only a small number of cases have been reported, the course, clinical manifestations, and visual prognosis have not been well defined. This study described the clinical features of 33 cases (33 eyes) of CCRAVO.

## 2. Materials and Methods

This retrospective study included 33 patients who had CCRAVO and were admitted to Hebei Eye Hospital, Hebei Province, China, between January 2013 and December 2017. Inclusion criteria were as follows: (1) acute unilateral visual loss; (2) manifestation of CRVO, e.g., flame-shaped or patchy retinal hemorrhage, dilated and/or tortuous retinal vein; (3) changes associated with CRAO, including ischemic retinal whitening, cherry-red spot in the macula, delayed filling of the retinal vessels, and prolonged retinal arteriovenous circulation on fundus fluorescein angiography (FFA).

The study adhered to the tenets of the Declaration of Helsinki and was approved by the institutional review board of the hospital. After informed consent was obtained from all participants, clinical data, such as age, gender, best-corrected visual acuity (BCVA), intraocular pressure, findings on fundus color photography and FFA, and optic coherent topography (OCT), were collected. A statistical description was generated with SPSS for Windows, version 18.0.

## 3. Results

The entity was unilateral in every case.  Table 1 summarizes the demographic characteristics of the 33 patients. Only approximately half of the patients had a history of systemic disease. Hypertension or diabetes was not especially common. One patient had Meniere's syndrome. One patient had rheumatism, and intracranial venous sinus thrombosis was noted after admission. One patient underwent cesarean section 13 days before presentation. One patient had recurrent oral ulcer in the previous two years, and Behçet's syndrome was suspected but not confirmed.

**Figure 1:**
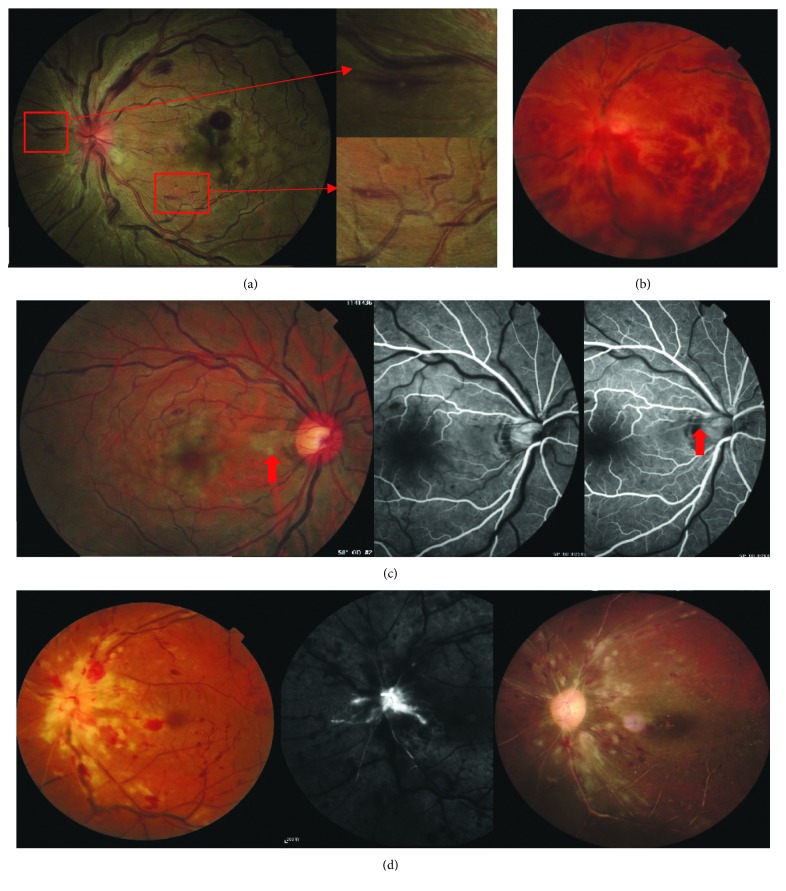
Fundus abnormalities at presentation. (a) Swelling optic disc with hyperemia, dilated and tortuous veins, mild hemorrhage, uneven macular ischemic whitening, atypical cherry-red spot, and Roth's spot. (b) Severe retinal hemorrhage, inferior vitreous hemorrhage, and perivenous sheathing along the superonasal branch vein. (c) Ischemic whitening with a clear border by the optic disc (red arrow). FFA showed delayed filling of the cilioretinal artery. (d) Optic disc edema without marked peripapillary CWS and completely occluded arteries in the nasal quadrant. FFA showed unfilled retinal vessels at the late stage. About one month after onset, a pale optic disc was seen and retinal vessels appeared as white lines.

All patients had a sudden decrease in visual acuity. As shown in Table 2, BCVA at presentation varied from no light perception (NLP) to 20/20. In 15 eyes (45.5%), BCVA was finger counting (FC) or less, but 4 eyes (12.1%) had BCVA of 20/60 or greater. The involved eyes had lower IOP (range, 10–24 mmHg; 15.0 ± 3.0 mmHg) than the contralateral eyes (range, 13–21 mmHg; 16.4 ± 2.3 mmHg), with a statistically significant difference (*t* = −2.273, *p*=0.03). Relative afferent pupillary defect (RAPD) was positive in 27 (81.8%) patients. Anterior chamber cells were observed in 3 patients, one of whom had marked anterior chamber cells complicated with recurrent oral ulcer, vitreous opacity and hemorrhage, and severe retinal hemorrhage (Figure 1(b)).

**Figure 2:**
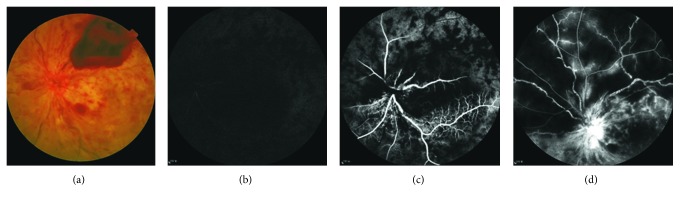
A woman had sudden visual loss for 2days. (a) Fundus photograph revealed ischemic retinal whitening, retinal hemorrhage, and preretinal hemorrhage. (b) FFA showed that the retinal arteries began to fill 15 s after injection of dye. (c) The retinal vein showed dye at 26 s. (d) Large areas of retinal capillary nonperfusion and severe vessel leakage developed.

**Figure 3:**
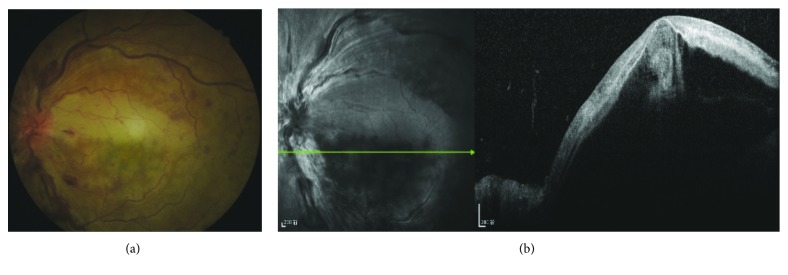
A woman had sudden visual decrease 13 days after cesarean section. (a) Fundus photograph showed dilated veins, mild hemorrhage, and uneven retinal ischemic whitening without a cherry-red spot. (b) OCT showed serous macular retinal detachment and hyperreflection of the inner retina.

Retinal hemorrhage in CCRAVO was similar to that in CRVO. In 15 eyes (45.5%), punctate, flame, or splinter-shaped hemorrhage was mainly restricted to the posterior pole (Figures 1(a) and 1(c)) and the periphery of the retina was relatively free of hemorrhage. In another 7 eyes, hemorrhage was marked in both the central and peripheral retina (7/33, 21.2%). One eye had preretinal hemorrhage at presentation (Figure 2(a)), and 1 patient had vitreous hemorrhage (Figure 1(b)). Optic disc hyperemia and edema (24/33, 72.7%), retinal venous dilation and tortuosity (32/33, 97.0%), and cotton wool spots (25/33, 75.8%) were common signs at the initial visit (Figure 1). The swelling optic disc in the involved eyes turned red as a result of hyperemia but did not protuberate high into the vitreous cavity. Nevertheless, a normal optic disc (2/33, 6.1%) or an optic disc with only hyperemia (4/33, 12.1%) or edema (3/33, 9.1%) was seen in a few cases. At presentation, all patients had retinal venous dilation and tortuosity except one who had vitreous injection of ranibizumab 2 weeks before presentation and showed narrowing retinal veins and arteries. Perivenous sheathing was seen in 2 eyes (Figure 1(b)). A total of 6 eyes had narrowing retinal arteries, 2 of which had completely occluded retinal arteries that appeared as white lines (Figure 1(d)). Retinal ischemic whitening of different degrees in the post pole was uneven, which made the cherry-red spot atypical in most eyes (Figure 2(a)). Milky-white opacity with a clear border adjacent to the retinal vascular arcade or in the papillomacular region was noted in 10 eyes (Figures 1(c) and 3(a)), and in 1 of these, the opacity was caused by cilioretinal artery occlusion instead of retinal arterial occlusion (Figure 1(c)). Additionally, Roth's spots were seen in 15 eyes (15/33, 45.5%) (Figure 1(a)).

All patients underwent FFA at presentation. Arm-to-retina time (ART) ranged from 7.0 to 27.6 s (17.1 ± 4.9 s). Only 2 eyes had normal ART (<11 s). The retinal vein did not show filling in the late stage of angiography (after 20 minutes) in 2 eyes (Figure 1(d)). In the other 31 eyes, retinal arteriovenous passage time (AVP) ranged from 2.7 to 44 s (12.1 ± 8.8 s) (Figures 2(b) and 2(c)). Twenty eyes underwent video recording at the early stage of FFA, and 14 of these showed oscillating filling. Capillary nonperfusion (CNP) was found in 21 eyes (63.6%) at presentation, 14 (42.4%) of which had CNP that was larger than 10 disc areas (Figures 2(c) and 2(d)).

OCT was performed on 20 eyes. The main abnormalities shown with OCT included retinal thickening (15 eyes, 75%), macular retinal serous detachment (11 eyes, 55%) (Figure 3(b)), hyperreflectivity in the inner retina (15 eyes, 75%), and macular cystoid edema (5 eyes, 25%).

All patients underwent emergent treatment, such as oxygen inhalation, retrobulbar injection of atropine, and intravenous administration of vasodilating agents. One patient was referred to the department of hematology because of anemia. Of the patients, 14 were treated with corticosteroid retrobulbarly or systemically.  Table 3 shows the changes in visual acuity and ophthalmoscopic exams 2 to 3 weeks after presentation. Of the involved eyes, 71.9% (23/32) had variable degrees of visual improvement but 15.6% (5/32) had visual deterioration. Two patients lost their fundus record. In the other 30 patients, more than half of the eyes showed alleviation of retinal ischemic whitening as well as optic disc hyperemia and edema. However, retinal hemorrhage increased in nearly half of the patients. Panretinal photocoagulation (PRP) was performed on 3 eyes in the early stage of the disease (within 1 month after onset). Follow-up ranged from 6 to 56 months. Seven patients were lost to follow-up. BCVA at the last follow-up is shown in Table 2. BCVA decreased in 7 eyes, and retinal hemorrhage was absorbed during months after onset. The optic disc turned pale in all eyes during follow-up (Figure 1(d)). Neovascularization occurred in only 4 eyes, including 2 eyes that had vitreous hemorrhage (VH) caused by retinal neovascularization and 2 eyes that had neovascular glaucoma (NVG). Eyes with VH had good visual acuity after emergent treatment (20/30 and 20/20, respectively). During follow-up, retinal hemorrhage increased and visual acuity decreased. Even though PRP was performed on these 2 eyes, retinal neovascularization and VH still occurred. Eyes that developed NVG initially present poor visual acuity (both FC), multiple CWSs, and completely occluded retinal arteries (Figure 1(d)). No improvement in visual acuity was seen after treatment, and NVG occurred 1 and 7 months, respectively, after onset.

## 4. Discussion

This study summarized the findings of a series of patients with CCRAVO. Most patients had severe visual damage, although some still had relatively good vision. Ophthalmoscopic fundus findings at presentation were characterized by manifestations of both CRAO and CRVO, including hyperemia and edema of the optic disc, tortuosity and/or dilation of the retinal vein, retinal hemorrhage, CWSs, and variable degrees of retinal ischemic whitening. FFA showed delayed filling of the retinal vessels, prolonged retinal arteriovenous circulation, and early retinal CNP. OCT showed retinal thickening, macular retinal serous detachment, and hyperreflectivity in the inner retina. In more than half of the eyes, visual acuity improved and signs associated with CRAO improved 2 to 3 weeks after presentation, whereas retinal hemorrhage increased in nearly half of the eyes. During follow-up, all eyes developed a pale optic disc. Approximately three-fourths of the eyes had visual acuity of 20/200 or below during follow-up. Although nearly two-thirds of the eyes had large areas of CNP at presentation, only a small number of eyes developed neovascularization.

Mean age of the patients in this study was 48.8 ± 14.1 years (range, 22–78 years), similar to that reported by Vallée et al. [10] and that of patients with combined central retinal vein and cilioretinal artery occlusion reported by Hayreh et al. [11]. However, the patients in this study were much younger than the patients reported by Brown et al. [12] and Schmidt [13]. They were also younger than the patients who had only CRVO [14] or CRAO [15].

Usually, the main symptom of CCRAVO is painless visual impairment. The degree or pattern of visual impairment is comparable to that of CRAO. Many reports have shown that CCRAVO frequently decreases visual acuity to FC and HM, or even to NLP [6, 12, 16, 17], but a few researchers have also shown that fairly good visual acuity can be maintained [10, 18–20]. In Brown's study [12], 87% (20/23) of eyes with CCRAVO had visual acuity between FC and NLP. Visual acuity remained FC or less in 14 of 15 eyes (93.3%) that underwent follow-up for 6 months. However, in this study, only 45.5% (15/33) of eyes had visual acuity of FC or less initially and 23.1% (6/26) had visual acuity of FC or less at final follow-up. In Vallee's study [10], final visual acuity was even better. Fewer than 20% of eyes had visual acuity of less than FC, whereas nearly half of the eyes had visual acuity of 20/25 or better. The degree and duration of ischemia caused by CCRAVO could account for the variation. In Brown's study, the patients were much older, more patients had systemic diseases or topical conditions, and more eyes had moderate or severe hemorrhage. However, the patients in Vallee's study seemed to have much milder retinal hemorrhage or edema, and much shorter interval between the onset and presentation, probably associated with better visual prognosis. The current study included more eyes, and the frequency of retinal damages, such as retinal hemorrhage or edema, was relatively even. So, in this study, some eyes maintained relatively good visual acuity, whereas others had poor visual acuity.

It has been reported that CRVO can cause a decrease in IOP in the involved eye [21]. This study also found this phenomenon in CCRAVO. The mechanism is unknown. The decrease in IOP may be related to release of cytokines by the retina as a result of ischemia or hemorrhage [21].

The diagnosis of CCRAVO is based on ophthalmoscopic fundus findings that include tortuosity and dilation of the retinal vein, swelling and hyperemia of the optic disc, retinal hemorrhage and ischemic whitening, a cherry-red spot, CWS, or Roth's spot. Abnormality of the optic disc and retinal vein is the most prominent sign in the early stage. Both in this study and in Brown's report [12], tortuosity and dilation of the retinal vein and swelling and hyperemia of the optic disc were frequently seen. Retinal hemorrhage varies in extent and degree in CCRAVO, similar to that in CRVO. Retinal ischemic whitening often distributes unevenly in the post pole, suggesting that ischemia varies in different parts of the retina. The macula is the most involved region, probably because of its poor tolerance to ischemia and vulnerable blood supply by paramacular small vessels. Slight ischemia may not cause significant retinal change. However, in some conditions, the ischemia is so severe in some regions that the involved retina turns milky-white with a clear border. A cherry-red spot is not always typical and may be indistinguishable (Figure 3(a)) as a result of uneven retinal ischemic whitening and macular edema caused by retinal vein occlusion. In time, retinal changes related to artery occlusion resolve earlier than those of CRVO. Finally, optic nerve atrophy develops.

FFA can evaluate the retinal circulation and the morphology of retinal vessels, providing useful information for eyes with CCRAVO. Delayed filling of the retinal vessels and prolonged retinal arteriovenous passage were common in CCRAVO. Some eyes in this study showed an oscillating filling pattern. This filling pattern means that the retinal artery fills for a variable distance during systole, but the filling retracts or stops during diastole. All of these signs suggest poor retinal perfusion and circulation. In addition, 63.6% of eyes (21/33) had CNP at presentation in this study, some of which was extensive. However, most eyes with CNP did not have neovascularization during follow-up.

Reports [1, 2, 12] have shown that CCRAVO can lead to retinal or iris neovascularization with poor visual prognosis. In Brown's study [12], 81% (17/21) of eyes developed rubeosis iridis during follow-up. Of the 3 eyes that underwent PRP, rubeosis iridis occurred in 2. By contrast, no eyes in Vallee's study [10] had neovascularization. In this study, only 4 eyes developed neovascularization. Of these, 2 had vitreous hemorrhage and the other 2 had neovascular glaucoma. Patients in Brown's study were older and had worse systemic or topical conditions. Their visual damage was also worse, suggesting more severe ischemia. All of these factors could be associated with ocular neovascularization. The eyes that developed neovascularization in this study showed some similarity. In the eyes that developed vitreous hemorrhage, visual acuity was relatively good after emergent treatment and deteriorated gradually thereafter with increasing hemorrhage. This process is similar to that of CRVO. Although PRP was performed, vitreous hemorrhage could not be prevented. In the other eyes with NVG, visual acuity was FC at presentation. Ophthalmoscopic evaluation showed marked CWSs and completely occluded retinal vessels that appeared as white lines on fundus photography or as unfilled retinal vessels on FFA. The eyes did not respond to emergent treatment. Rubeosis iridis developed during follow-up. This neovascularization is similar to that induced by CRAO, which is associated with extraordinary prolonged retinal vessel filling and failure to gain reperfusion [22, 23].

The mechanism of CCRAVO has not been well studied. CRVO may play a more critical role than CRAO. Brown's study [12] and this study both found cases in which CCRAVO followed CRVO, suggesting CRAO could be a complication of CRVO. Previous reports showed that CRVO can occur in combination with central or branch retinal artery occlusion or cilioretinal artery occlusion. In this case series, there is also a case of CRVO in combination with central retinal and cilioretinal artery occlusion. The extent of retina involved by vein occlusion is always larger than or equal to that involved by artery occlusion. Artery occlusion could be secondary to CRVO, which increases the intraluminal pressure of the retinal capillary bed, and the pressure is transmitted to the retinal artery to decrease or even stop the blood flow. Furthermore, this study showed that when ischemic whitening, together with optic disc hyperemia and swelling, resolved in more than half of the involved eyes during the first 2 to 3 weeks after presentation, retinal hemorrhage continued to deteriorate in nearly half of the eyes. CRVO persisted much longer than CRAO. When CRVO occurs, there may be simultaneous abnormal retinal artery perfusion [24]. Optic disc swelling and retinal edema related to CRVO may further compress the retinal arteries, narrowing and possibly occluding them. This may be the reason why there is narrowing of the arteries with CCRAVO.

## 5. Conclusion

In summary, CCRAVO is a sight-threatening entity. The involved eye shows changes associated with both CRAO and CRVO simultaneously at presentation, but CRVO may play a more important role in the development of CCRAVO. CRAO could resolve sooner than CRVO. A pale optic disc develops at last, and only a small number of eyes develop neovascularization.

## Figures and Tables

**Table 1:** Demographic features and history.

Characteristic	Total (*n* = 33)
Male, *n* (%)	20 (60.6%)
Onset age	
Range (yr)	22–78
Mean (yr, mean ± SD)	48.8 ± 14.1
Median (yr)	51.0
Systemic history	
Hypertension	10 (30.3%)
Diabetes	2 (6.1%)
Anemia	3 (9.1%)
Other	4 (12.1%)
Interval between onset and presentation	7 hr–2 mo
≤1 day	8 (24.2%)
1–3 days	8 (24.2%)
3–7 days	9 (27.3%)
7–14 days	6 (18.2%)
>14 days	2 (6.1%)

**Table 2:** BCVA at presentation, 2 to 3 weeks after presentation, and at last follow-up.

BCVA	At presentation	Two to 3 weeks after presentation	Last follow-up
≥20/60	4 (12.1%)	7 (21.9%)	6 (23.1%)
20/200–20/60	3 (9.1%)	6 (18.8%)	1 (3.8%)
20/400–20/200	0	4 (12.5%)	4 (15.4%)
20/2000–20/400	11 (33.3%)	11 (33.3%)	9 (34.6%)
FC, LP, or NLP	15 (45.5%)	4 (12.5%)	6 (23.1%)
Total (eyes)	33	32	26

**Table 3:** Changes 2 to 3 weeks after presentation.

	BCVA	Optic disc hyperemia or edema	Retinal ischemic whitening	Retinal hemorrhage
Improved	23 (71.9%)	17 (56.7%)	24 (80.0%)	9 (30.0%)
No change	4 (12.5%)	10 (33.3%)	3 (10.0%)	6 (20.0%)
Decreased	5 (15.6%)	3 (10.0%)	3 (10.0%)	15 (50.0%)
Total (eyes)	32	30 eyes	30 eyes	30 eyes

## Data Availability

The data used to support the findings of this study are available from the corresponding author on request.

## References

[1] Parchand S. M., Vijitha V. S., Misra D. P. (2017). Combined central retinal artery and vein occlusion in lupus. *BMJ Case Reports*.

[2] Nishiguchi K. M., Ito Y., Terasaki H. (2013). Bilateral central retinal artery occlusion and vein occlusion complicated by severe choroidopathy in systemic lupus erythematosus. *Lupus*.

[3] Kahloun R., Jelliti B., Abroug N. (2016). Combined central retinal artery occlusion and central retinal vein occlusion secondary to Behcet’s disease. *Journal Français d’Ophtalmologie*.

[4] Sánchez-tocino H., García-layana A., Salinas-alamán A., Alcalde-navarrete J. M., Panizo-santos A., Martínez-monge R. (2004). Central retinal vascular occlusion by orbital pseudotumor. *Retina*.

[5] Shukla D., Mohan K. C., Rao N., Kim R., Namperumalsamy P., Cunningham E. T. (2004). Posterior scleritis causing combined central retinal artery and vein occlusion. *Retina*.

[6] Vasavada D., Baskaran P., Ramakrishnan S. (2017). Retinal vascular occlusion secondary to retrobulbar injection: case report and literature review. *Middle East African Journal of Ophthalmology*.

[7] Bouraoui R., Mghaieth F., Bouladi M., Limaiem R., Maamouri R., El Matri L. (2016). Combined central retinal arterial and venous occlusion after ocular contusion. *Journal Français d’Ophtalmologie*.

[8] Parchand S. M. (2016). Combined central retinal vein and branch retinal artery occlusion in hyperhomocysteinaemia. *BMJ Case Reports*.

[9] Salazar Méndez R., Fonollá Gil M. (2014). Edema de papila y obstrucción de arteria y vena central de la retina como manifestación inicial de una recaída leucémica. *Archivos de la Sociedad Española de Oftalmología*.

[10] Vallée J.-N., Paques M., Aymard A. (2002). Combined central retinal arterial and venous obstruction: emergency ophthalmic arterial fibrinolysis. *Radiology*.

[11] Hayreh S. S., Fraterrigo L., Jonas J. (2008). Central retinal vein occlusion associated with cilioretinal artery occlusion. *Retina*.

[12] Brown G. C., Duker J. S., Lehman R., Eagle R. C. (1993). Combined central retinal artery-central vein obstruction. *International Ophthalmology*.

[13] Schmidt D. (2013). Comorbidities in combined retinal artery and vein occlusions. *European Journal of Medical Research*.

[14] Hayreh S. S., Podhajsky P. A., Zimmerman M. B. (2011). Natural history of visual outcome in central retinal vein occlusion. *Ophthalmology*.

[15] Hayreh S. S., Zimmerman M. B. (2005). Central retinal artery occlusion: visual outcome. *American Journal of Ophthalmology*.

[16] Hwang H. S., Kang S. (2012). Combined central retinal vein and artery occlusion in systemic lupus erythematosus patient. *Retinal Cases & Brief Reports*.

[17] Akhlaghi M., Abtahi-Naeini B., Pourazizi M. (2018). Acute vision loss in systemic lupus erythematosus: bilateral combined retinal artery and vein occlusion as a catastrophic form of clinical flare. *Lupus*.

[18] Chang P.-C., Chen W.-S., Lin H.-Y., Lee H.-M., Chen S.-J. (2010). Combined central retinal artery and vein occlusion in a patient with systemic lupus erythematosus and anti-phospholipid syndrome. *Lupus*.

[19] Sowka J. W., Vollmer L. A., Au M. (2015). Atypical retinal vaso-occlusion with structural and functional resolution. *Optometry and Vision Science*.

[20] Lima V. C., Prata T. S., Landa G., Yannuzzi L. A., Rosen R. B. (2010). Intravitreal triamcinolone and bevacizumab therapy for combined papillophlebitis and central retinal artery occlusion. *Retinal Cases & Brief Reports*.

[21] Hayreh S. S., Zimmerman M. B., Beri M., Podhajsky P. (2004). Intraocular pressure abnormalities associated with central and hemicentral retinal vein occlusion. *Ophthalmology*.

[22] Jung Y. H., Ahn S. J., Hong J.-H. (2016). Incidence and clinical features of neovascularization of the Iris following acute central retinal artery occlusion. *Korean Journal of Ophthalmology*.

[23] Duker J. S., Brown G. C. (1988). Iris, neovascularization associated with obstruction of the central retinal artery. *Ophthalmology*.

[24] Hayreh S. S., Van Heuven W. A., Hayreh M. S. (1978). Experimental retinal vascular occlusion. *Archives of Ophthalmology*.

